# Structure–Activity Relationship Assessment of Sophorolipid Ester Derivatives against Model Bacteria Strains

**DOI:** 10.3390/molecules26103021

**Published:** 2021-05-19

**Authors:** Filbert Totsingan, Fei Liu, Richard A. Gross

**Affiliations:** 1 Department of Chemistry and Chemical Biology, Rensselaer Polytechnic Institute, Troy, NY 12180, USA; 2 Center for Biotechnology and Interdisciplinary Studies (CBIS), Rensselaer Polytechnic Institute, Troy, NY 12180, USA; l.feigz@gmail.com

**Keywords:** sophorolipids, antimicrobial activity, structure–activity relationship, selectivity, bacterial resistance

## Abstract

Sophorolipids (SLs) are glycolipids that consist of a hydrophilic sophorose head group covalently linked to a hydrophobic fatty acid tail. They are produced by fermentation of non-pathogenic yeasts such as *Candida Bombicola*. The fermentation products predominantly consist of the diacetylated lactonic form that coexists with the open-chain acidic form. A systematic series of modified SLs were prepared by ring opening of natural lactonic SL with *n*-alkanols of varying chain length under alkaline conditions and lipase-selective acetylation of sophorose primary hydroxyl groups. The antimicrobial activity of modified SLs against Gram-positive human pathogens was a function of the *n*-alkanol length, as well as the degree of sophorose acetylation at the primary hydroxyl sites. Modified SLs were identified with promising antimicrobial activities against Gram-positive human pathogens with moderate selectivity (therapeutic index, TI = EC_50_/MIC*_B. cereus_* = 6–33). SL-butyl ester exhibited the best antimicrobial activity (MIC = 12 μM) and selectivity (TI = 33) among all SLs tested. Kinetic studies revealed that SL-ester derivatives kill *B. cereus* in a time-dependent manner resulting in greater than a 3-log reduction in cell number within 1 h at 2×MIC. In contrast, lactonic SL required 3 h to achieve the same efficiency.

## 1. Introduction

Bacterial resistance has spread worldwide and is now causing a global health crisis, which requires a global action plan [1]. For example, it is reported that more than 2.8 million antibiotic-resistant infections occur in the United States each year, and more than 35,000 people die as a result [2], while at least 37,000 people die in the EU each year as a direct consequence of healthcare-related infections [3]. One major concern is that the number of approved antibiotics capable of combating bacterial resistance has consistently dropped over the years [4], thus suggesting an urgent need for new drugs that will address this worldwide problem. In drug discovery and development, it is essential to use strategies that reduce the likelihood of new therapeutics being potential targets for the development of pathogen resistance. One of the strategies to circumvent such outcomes is to focus on membrane-active drug development. This approach is favorable since it leads to rapid pathogen eradication, multi-target effects, and activity against slow-growing bacteria [5].

**Figure 1:**
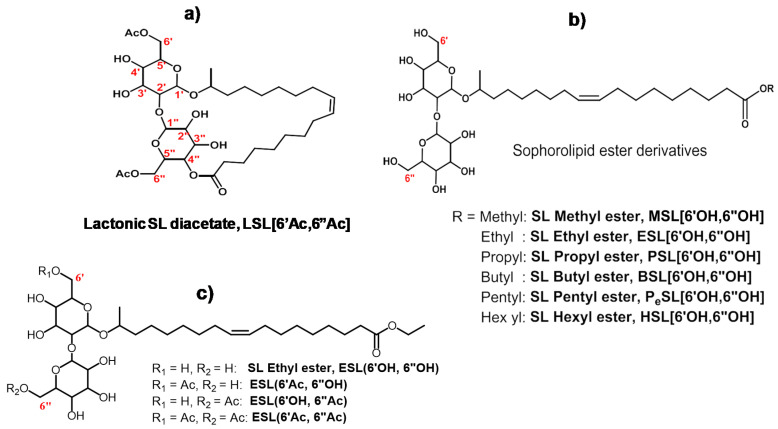
Chemical structures of (**a**) lactonic SL, (**b**) SL esters with increasing lengths, and (**c**) SL-ethyl ester with different degrees of acetylation.

Sophorolipids (SLs) are a family of glycolipids produced by fermentation of non-pathogenic yeasts such as *Candida Bombicola* [6,7]. They consist of a hydrophilic sophorose head covalently linked to a hydrophobic fatty acid tail. The fermentation products are predominantly lactonic forms with various degrees of acetylation on the sophorose head (Figure 1a) that coexist with opened chain acidic forms [8,9,10]. Modified SLs possess great potential as emulsifying agents [10,11,12] and were also found to exhibit a broad range of biological properties, including antibacterial [13], antiviral [14], anticancer [15,16], and anti-inflammatory [17] properties; spermicidal agents [14]; and modulators to control the severity of sepsis [18]. Despite their promising bioactivities, there remains a gap in our understanding of natural and modified SL structure–activity relationships. Efforts to improve the performance of natural SLs include varying the hydrophobic tail length or the sophorose structure during the biosynthetic processes [19,20] and genetic engineering to prepare other structural variants [21]. However, these approaches are often limited by a narrow range of structure variations that are produced in substantially lower yields than that produced by the wild-type organism. One promising and practical alternative is the use of chemo-enzymatic synthetic approaches to prepare a broad range of SL derivatives from natural lactonic SLs [22,23,24,25]. For instance, SL esters have been synthesized by ring opening of lactonic SLs with various alcohols under alkaline conditions [22]. Selective acylation on the two-primary hydroxyl groups of the sophorose unit has also been carried out by lipase-catalyzed reactions [24]. The change in surface tension and emulsifying properties of SL esters with increasing chain length has also been investigated [10,11,12,23]. Thus, the chemo-enzymatic approach provides a wide range of modified SLs to interrogate SL structure–activity relationships.

In this study, a family of SL esters (Figure 1b) with increasing chain length and degree of acetylation of the sophorose moiety (Figure 1c) were synthesized and studied for their potential as antimicrobial agents. Structure–activity relationships were investigated, and the ability of these SL derivatives to prevent resistance to human pathogens was evaluated. To gain insights into their mechanism of action, their membrane depolarization capability was assessed. The cumulative results presented herein are promising and provide a foundation that will guide further development of modified SL antimicrobials that are increasingly potent, and exhibit broad-spectrum activity and higher selectivity towards microbial pathogens.

## 2. Results and Discussion

### 2.1. In Vitro Antibacterial Efficacy and Structure–Activity Relationship of SL Derivatives

To assess the structure–activity relationship (SAR) of SLs, a family of SL-esters was synthesized from natural lactonic SL by transesterification using sodium alkoxides of different lengths (Figure 1b). The antibacterial activity of these SL derivatives was evaluated by testing the minimum inhibitory concentration (MIC) against *E. coli* and several Gram-positive bacteria. The MICs of SL-methyl ester against *B. cereus*, *B. subtilis*, *S. aureus,* and *L. innocua* were estimated to be 50, 50, 200, 100 μM, respectively (Table 1). As the length of the ester moiety increases, the MIC generally decreases, reaching the lowest values with SL-butyl ester against all tested pathogens (12, 12, 100, and 12 μM against *B. cereus*, *B. subtilis*, *S. aureus,* and *L. innocua,* respectively). The SL-butyl ester exhibited superior or equivalent activity relative to natural LSL for *B. cereus, B. subtilis,* and *L. innocua,* and to the control antibiotic, streptomycin. SL-pentyl ester was only active against *B. cereus* and *L. innocua*. Further increase in the ester chain length (n > 5, i.e., SL-hexyl ester) did not show any antibacterial activity up to 200 μM. This loss of activity likely indicated that longer carbon chain esters failed to confer sufficient hydrophilicity to maintain a good hydrophilic–hydrophobic balance. Furthermore, LSL and all the SL-ester derivatives exhibited poor activity against *E. coli* (MIC > 200 μM). The lack of activity against *E. coli* is likely due to the presence of an outer membrane that acts as an additional barrier, as well as other defense pathways that are not present in Gram-positive bacteria [26]. LSL selective activity against Gram-positive strains was previously reported by Kim et al. [27].

The degree of acetylation was reported to substantially affect several SL properties, including oil phase emulsification as well as antibacterial, spermicidal, and anti-HIV activities [14,23,28]. To assess the extent that the degree and site of acetylation have on SL antibacterial efficacy, the MICs of the SL-ethyl ester acetylated at the 6′- and/or 6″-positions of the sophorose unit were determined, and the results are summarized in Table 2. For the mono-acetylated SL-ethyl ester, the site of acetylation did not influence the MIC against *B. subtilis*, *S. aureus,* and *L. innocua*. However, monoacetylation decreased the MIC against *B. subtilis*, *S. aureus,* and *L. innocua* from 50 to 25 μM. While increasing the degree of acetylation so that both the 6′- and 6″-positions were acetylated decreased the MIC to 12 μM for *B. Cereus*, it did not exhibit any antibacterial activity against *B. subtilis*, *S. aureus,* or *L. innocua*. These results indicate that SL-ester chain length, sophorose unit degree of acetylation, and cell morphology all play an important role in antibacterial activity. These results are consistent with a previous study on the effect of acetylation degree on the antibacterial activity of LSL [28].

### 2.2. Time–Kill Studies

**Figure 2:**
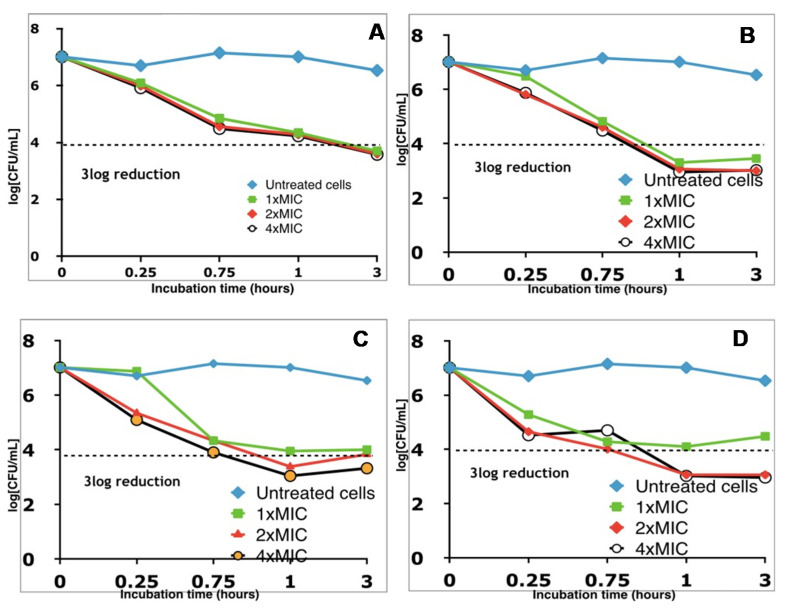
Time–kill study of sophorolipid ester derivatives against *B. cereus*: (**A**) lactonic SL; (**B**) SL-methyl ester; (**C**) SL-propyl ester; (**D**) SL-butyl ester.

To further investigate if the growth inhibition was associated with cell death, a killing kinetic study was conducted on *B. cereus. B. cereus* inocula (10^7^ CFU/mL) were exposed to SL derivatives at 1×MIC, 2×MIC, and 4×MIC in PBS buffer at pH 7.4. At 15 min, 45 min, 1 h, and 3 h, aliquots were spread onto agar plates for cell counting. As shown in Figure 2, all SL derivatives tested exhibited a time-dependent bactericidal activity. Cells treated with 2×MIC and 4×MIC of SL-esters displayed a >3 log reduction (>99.9% reduction) in cell number within 1 h (Figure 2B–D), while lactonic SL reached the same level of killing in about 3 h (Figure 2A). This result shows that, at least with *B. cereus*, natural lactonic SL is less effective in killing this bacterium than the modified SL-ester derivatives, thus showing the benefit of molecular editing in enhancing the killing efficiency of sophorolipids.

### 2.3. Membrane Depolarization Assay

**Figure 3:**
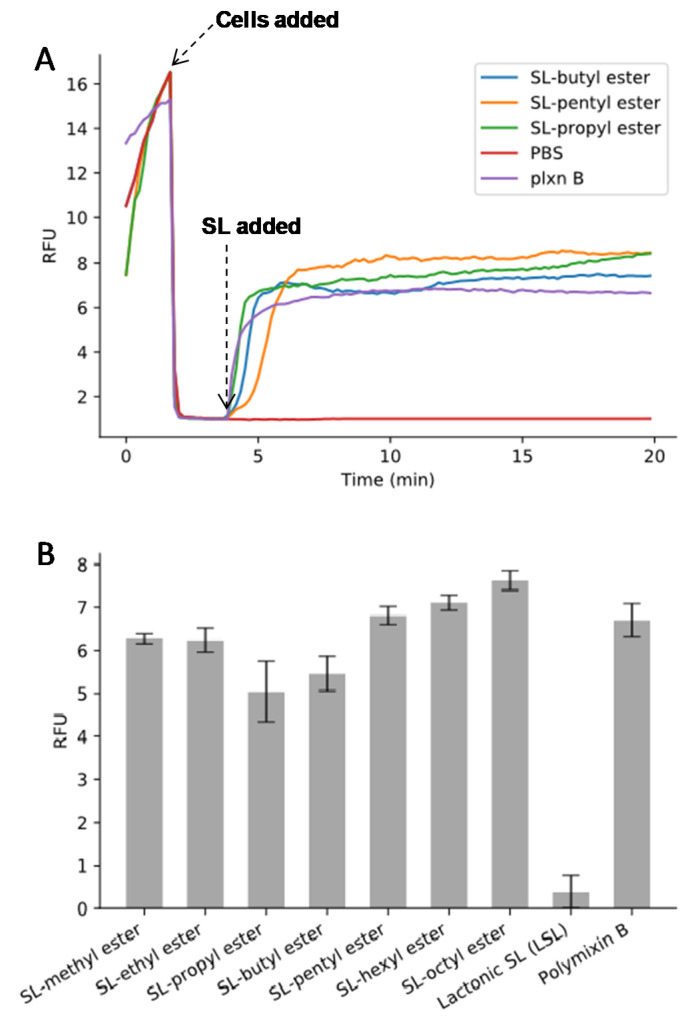
Dye leakage assay of SL esters: (**A**) time dependence of fluorescence response; (**B**) endpoint fluorescence intensity measured at t = 60 min. Polymyxin B (plxn B) was used as a positive control.

To probe the role of LSL and the SL-esters as membrane-active agents, dye leakage experiments were carried out. Polymyxin B, a well-known membrane-active agent, was used as a positive control. Polymyxin B is a cyclic peptide with long, hydrophobic tails that can insert into cell membranes causing cell lysis. First, time-course membrane-depolarizing studies were conducted (Figure 3A). At time 0 min, the fluorescent probe, diS-C3-(5), exhibited a strong fluorescence signal. Upon addition of *B*. *cereus* cells at 2 min, a rapid decay in fluorescence intensity was observed due to self-quenching caused by migration of the probe into membranes. Subsequent addition of the SL-esters at 4 min led to a sharp increase in the fluorescence signal, thus indicating enhanced permeability of the cell membrane driven by the presence of the SL derivative. This is consistent with the time–kill studies that showed rapid bactericidal activities of SLs on *B. cereus*. Endpoint fluorescence data were recorded at t = 60 min and are displayed in Figure 3B. The results revealed that all modified SL-esters have a high ability to enhance membrane permeability to an extent that was similar to the positive control (polymyxin B). In contrast, LSL exhibited a non-significant ability to enhance cell membrane permeability. This suggests that the killing mechanism of LSL differs from that of SL-esters. The results also showed that SL esters with longer hydrophobic carbon chains (C6–C8) have good cell membrane permeability, despite that they exhibited no antimicrobial activity. This suggests that while membrane permeability is implicated in SL-ester activity, other mechanistic pathways are involved.

### 2.4. In Vitro Cytotoxicity

A potential antimicrobial candidate must selectively kill pathogen cells without harming the host mammalian cells. To test the ability of SLs to effectively discriminate between bacteria and mammalian cells, a cytotoxicity assay was previously reported by our team [17]. The SL-methyl ester exhibited the lowest toxicity with no significant cell reduction at 400 µM after 16 h incubation. SL-ethyl, propyl, and butyl esters exhibited low to no cytotoxicity up to 100–200 µM, which is much higher than MICs against most strains tested herein (12–50 µM for *B. cereus*, *B. subtilis,* and *L. innocua*). The SL-octyl ester was the most toxic of the SL-ester series, possibly due to its high hydrophobicity and affinity to cell membranes. Similarly, previous work with antimicrobial peptides (AMPs) showed that increasing their hydrophobicity enhanced their binding affinity to mammalian cell membranes [29,30]. In comparison to the ester series, lactonic SL was found to exhibit the highest cytotoxicity, with roughly 50% reduction in cell viability occurring at 50 µM, thus suggesting that molecular editing not only improved its antimicrobial activity but also lowered its toxicity against mammalian cells. The therapeutic index (TI) is a parameter commonly used to determine potential drug selectivity. TI values were estimated as EC_50_/MIC*B. cereus*, where EC_50_ is the concentration that corresponds to 50% cell death after 16 h incubations. Table 1 shows that molecular editing largely increased the TI relative to the natural parent lactonic SL. SL butyl ester exhibited the highest TI, which was 16× higher than that of lactonic SL. Generally, membrane-active agents are not considered for systemic administration directly, because of relatively low selectivity. Thus, designing suitable formulations and selecting suitable dose ranges are important in drug development. One successful clinical example is amphotericin B that was applied as a liposomal formulation, which largely decreased the side effect caused by high toxicity [31].

## 3. Materials and Methods

### 3.1. Materials and Reagents

Novozym 435 was obtained from Novozymes (Copenhagen, Denmark). Lipase PS-C was purchased from Sigma-Aldrich (Milwaukee, WI, USA). Sodium metal (large pieces in kerosene) was purchased from Fisher Scientific (Pittsburgh, PA, USA). 3,3′-Dipropylthiadicarbocyanine iodide (diS-C3(5)) and polymyxin B were purchased from Sigma-Aldrich. Tetrahydrofuran (THF), chloroform (CHCl_3_), methanol (CH_3_OH) and dimethyl sulfoxide (DMSO) were purchased from Fisher Scientific (Pittsburgh, PA, USA). All bacteria culture media and agars were purchased from Sigma-Aldrich (Milwaukee, WI, USA). All other chemicals and solvents were purchased in the highest available purity from Sigma-Aldrich and were used without further purification.

### 3.2. SL Synthesis

Lactonic SL and SL esters were prepared with good purity (>95%) following the experimental procedures previously reported by us [6,7,22,23,24]. In brief, in a round-bottom flask equipped with a reflux condenser, dry diacetyl lactonic SL (29 mmol) was dissolved in the corresponding alcohol (40 mL). To this mixture, 10 mL of sodium alkoxide was slowly added, which was prepared separately by adding a small piece of Na metal (170 mg, 0.25 eq) in the corresponding alcohol. The resulting mixture was refluxed (or heated at 100 °C) for 3 h, and the reaction was monitored by TLC (CHCl_3_/CH_3_OH, staining solution: cerium ammonium molybdate (CAM)). Upon completion, the reaction mixture was cooled to room temperature, acidified with 1 M HCl or glacial acetic acid to pH 4–5, and concentrated by rotoevaporation. Precipitation in ice-cold water (0.5 L), followed by filtration and dryness under vacuum, yielded the desired product as an off-white solid (80–85%). Longer alkyl chain SL esters (C4–C8) were further purified by flash chromatography. The SL esters were characterized by ^1^H NMR and LC-MS.

Selective acetylation of the sophorose primary hydroxyls was achieved using two different immobilized lipases (lipase PS-C and Novozym 435). To a solution of SL-ethyl ester (4.0 g; 6.14 mmol) and vinyl acetate (5 or 10 eq) in dry THF (50 mL), either lipase PS-C (1.23 g) for 6′′-acetylation or Novozym 435 (1.5 g) for 6′- and 6′,6″-acetylation was added. The resulting mixture was stirred at 40 °C under an N_2_ atmosphere, and the reaction progress was monitored by TLC. Upon completion, the enzyme was filtered out, followed by washing with THF (twice). After removal of solvent, the residue was subjected to silica gel column chromatography using chloroform/methanol as eluent (gradient: from 95/5 to 80/20) to afford the final products as off-white solids (75–85%).

### 3.3. Bacteria Strain Culture and Antibacterial Testing

Bacteria strains *Staphylococcus aureus* (ATCC 33807), *Bacillus cereus* (ATCC 4342), *Bacillus subtilis* (ATCC 21332), *Listeria innocua* (ATCC 33090), and *Escherichia coli* (ATCC 53323) were provided by Ravi Kane’s lab at Rensselaer Polytechnic Institute. Mueller–Hinton broth (MHB) was used for the *Staphylococcus aureus* culture, while tryptic soy broth (TSB) was used for the other strains. All strains were grown to the stationary phase for 24 h at 37 °C with agitation at 220 rpm. After overnight incubation, cultures were diluted to 5 × 10^6^ CFU/mL prior to use.

The minimum inhibitory concentration (MIC) was determined using the microdilution technique. Modified sophorolipid (SL) derivatives were dissolved in DMSO to make 2 M stock solutions. A dilution series was made by diluting the stock solution to a range of concentrations from 200 µM to 4 µM and a final volume of 100 μL in 10% DMSO/phosphate-buffered saline (PBS) in each well of a 96-well plate. Bacterial inocula (100 μL, 5 × 10^6^ CFU/mL) were added to each well, and the final DMSO concentration was 5%. Wells containing the same volumes of DMSO/PBS buffer and culture medium, with and without bacteria, were used as positive and negative controls, respectively. Streptomycin was used for comparison. The plate was then incubated with shaking for 16–30 h at 37 °C to ensure that cultures had reached stationary phase growth and the absorbance readings were recorded at a wavelength of 600 nm using an absorbance microplate reader. Absorbance data from 16–30 h were used to calculate the percent inhibition for each test condition. MIC was recorded as the lowest concentration of drug that inhibited more than 90% of bacterial growth. All assays were carried out in triplicate.

### 3.4. Time–Kill Studies

The bactericidal activity of modified SLs against *Bacillus cereus* was assessed by incubating 1 mL of microbial suspensions (~10^7^ CFU/mL) and SLs (1×MIC, 2×MIC, 4×MIC) in 5% DMSO/PBS buffer at room temperature with constant shaking at 200 rpm. Aliquots of 20 µL were withdrawn periodically at t = 0, 0.25, 0.75, 1, and 3 h, and a series of 10× dilutions were made before spreading onto nutrient agar plates. The agar plates were then incubated at 37 °C for 12 h. The killing rate was measured by comparing colonies grown on each agar plate with colony counts from the untreated control.

### 3.5. Cytotoxicity Study

The activity of SL derivatives against M1 activated macrophages (AMOs) obtained after the stimulation of Raw 254.7 (ATCC) murine macrophages with LPS (lipopolysaccharide; 1 µg/mL) for 4 days was assessed following the procedure reported in the literature by us [17].

### 3.6. Dye Leakage Experiment

The disruption of bacterial membranes after treatment with LSL and SL-ester derivatives was tested using a fluorescence probe, diS-C3(5), which can be quenched by the electrically polarized membrane. *B. Cereus* grown to mid-log phase was adjusted to 2.5 × 10^6^ CFU/mL in fresh MHB. Subsequently, 100 μL of cell suspensions were mixed with 50 μL of LSL and SL-ester solutions (40 μg/mL, 58–60 μM) and 50 μL of diS-C3(5) (190 μg/mL, 348 μM), followed by incubation at 37 °C for 1 h. Polymyxin B (100 μg/mL, 77 μM), a well-characterized membrane-depolarized peptide, and PBS were used as positive and negative controls, respectively. Fluorescence data were recorded at 670 nm with excitation at 622 nm on Molecular Devices SPECTRAmax Plus 384 UV-VIS High Throughput Microplate Spectrophotometer and normalized against the negative control.

## 4. Conclusions

In summary, we designed and synthesized a library of SLs that exhibit promising antimicrobial activities against Gram-positive human pathogens with moderate selectivity (TI = 6–33). SL-butyl ester exhibited the best selectivity among all the SLs tested. Kinetic studies revealed that SL-ester derivatives kill *B. cereus* in a time- and concentration-dependent manner resulting in greater than a 3-log reduction in cell number within 1 h at 2×MIC. In contrast, LSL required 3 h to cause a 3-log reduction in *B. cereus*. The results of a depolarization assay performed using diS-C3-(5) as a fluorescent probe revealed that, except for LSL, all structurally edited SLs show a strong ability to enhance membrane permeability. Longer carbon chain SL esters exhibit a strong depolarizing effect despite their inability to kill bacterial cells. In contrast, LSL failed to enhance membrane permeability despite its good antimicrobial activity. These results strongly suggest that, while membrane disruption may be involved in LSL and SL-ester antimicrobial activity, additional pathways are involved.

## Figures and Tables

**Table 1:** Effect of SL-ester chain length on minimum inhibitory concentrations (MICs).

SLs	MIC (µM)					TI
	*B. cereus* ^1^	*B. subtilis* ^1^	*S. aureus* ^1^	*L. innocua* ^2^	*E. coli* ^1^	
Lactonic SL ^3^	25	12	25	50	>200	2
SL-methyl ester	50	50	200	100	>200	>8
SL-ethyl ester	50	50	200	50	>200	6
SL-propyl ester	25	25	100	25	>200	16
SL-butyl ester	12	12	100	12	>200	33
SL-pentyl ester	12	>200	>200	12	>200	17
SL-hexyl ester	>200	>200	>200	>200	>200	-
SL-octyl ester	>200	>200	>200	>200	>200	-
Streptomycin	12	12	3	24	12	-

^1^ Incubation time: 16 h. ^2^ Incubation time: 30 h. ^3^ Natural component used to prepare SL esters. Therapeutic index (TI) was calculated as EC_50_ macrophage/MIC*_B. Cereus_*. EC_50_ was determined as concentration causing 50% killing of macrophage after 16 h.

**Table 2:** Effect of degree of acetylation on minimum inhibitory concentrations.

SLs	MIC (µM)			
	*B. Cereus* ^1^	*B. Subtilis* ^1^	*L. innocua* ^2^	*E. coli* ^1^
ESL-(6′OH, 6″OH) ^3^	50	50	50	>200
ESL-(6′Ac, 6″OH)	25	25	25	>200
ESL-(6′OH, 6″Ac)	25	25	25	>200
ESL-(6′Ac, 6″Ac)	12	>200	>200	>200

^1^ Incubation time: 16 h. ^2^ Incubation time: 30 h. ^3^ ESL: SL-ethyl ester.

## Data Availability

Data available in a publicly accessible repository.

## Sample Availability

Samples of the compounds are available from the authors.
